# Reversible Non‐Stick Behaviour of a Bacterial Protein Polymer Provides a Tuneable Molecular Mimic for Cell and Tissue Engineering

**DOI:** 10.1002/adma.201304645

**Published:** 2014-03-13

**Authors:** Ana I. Roque, Andrei Soliakov, Mark A. Birch, Sion R. Philips, Deepan S. H. Shah, Jeremy H. Lakey

**Affiliations:** ^1^ Institute for Cell and Molecular BiosciencesNewcastle UniversityNewcastle upon TyneNE2 4HHUnited Kingdom; ^2^ Institute for Cell and Molecular BiosciencesNewcastle UniversityNewcastle upon TyneNE2 4HHUnited Kingdom; ^3^ Institute for Cellular MedicineNewcastle UniversityNewcastle upon TyneNE2 4HHUnited Kingdom; ^4^ Orla Protein Technologies LtdInternational Centre for LifeTimes SquareNewcastle upon TyneNE1 4EPUK

Regenerative medicine demands the recreation of complex cell–cell and cell–matrix interactions observed in vivo^1^ and has led to the development of artificial biomaterials to mimic the protein network in the extracellular matrix (ECM).^2^ Future developments would benefit from an economic supply of protein polymers which closely match the molecular structure of the natural material. The polymeric Caf1 protein, from the plague bacterium *Yersinia pestis*, forms an enveloping hydrogel whose role is to inhibit interactions with host cells.^4^ Furthermore, it shares a 3D structure with the largest class of human extracellular proteins. Here we show that recombinant Caf1 is a highly adaptable scaffold for a synthetic biology route to protein polymer engineering. It is robust, resisting both thermal and proteolytic degradation and when purified it retains its biological ability to prevent mammalian cell attachment; three advantageous properties difficult to design into a protein de novo. We then reverse the “non‐stick” phenotype by inserting a cell adhesion motif, express mixed polymers of different subunits and form hydrogels using a simple cross‐linker. Such animal free proteins, economically produced in *E. coli*, offer a new family of tissue culture materials.

Gram‐negative bacteria often form protein polymers on their surfaces via the chaperone‐usher (CU) pathway,^5^ so named because monomeric subunits, secreted into the bacterial periplasm, are initially stabilized by a specific chaperone protein.^6^ Subsequently this binary complex interacts with an outer membrane “usher” protein^7^ that provides a channel through to the extra‐cellular surface. The nascent polymer grows out through this usher and across the outer membrane by the addition of monomeric subunits to its periplasmic end. Each monomer donates a single beta‐strand to the preceding monomer thus joining the subunits by a strong but non‐covalent link. Many CU proteins of pathogenic bacteria are so called adhesins, involved in binding to host cells,^8^ but the bacterium *Yersinia pestis*, the etiologic agent of the bubonic plague, produces a unique form of CU polymer. After being injected into a warm blooded host via a flea bite,^9^ a temperature sensitive promoter expresses the *caf1* gene to produce a thick polymeric CU hydrogel coating on the surface of the cell. This efficiently resists cell interactions and, by acting as a kind of anti‐adhesin, inhibits macrophage attacks.^4^

The formation of the *Y. pestis* Caf1 protein has been described by a series of elegant papers from Knight and co‐workers who solved the high resolution structures of the chaperone (Caf1M‐Caf1) and also revealed the energetics of the polymer formation.^10^ We recently determined, by electron microscopy, the structure of Caf1 and revealed for the first time the conformational flexibility and large size of these poly­mers.^12^ They were up to 2 μm in length consisting of 800 monomers with molecular weights of up to 11 MDa. Usefully, these show no aggregation (apart from some inevitable knots) and remain soluble at >50 mg mL^−1^. Furthermore the polymers are stable up to 90 °C,^13^ protease resistant and easily purified.^14^

Models of Caf1 structure^12^ and the structure of the similar Saf protein^16^ predict it to be a polymer of immunoglobulin like domains (**Figure** **1**A and B). As such it resembles the predominant family of extracellular proteins in humans (∼3% of human protein‐coding genes) which includes fibronectin type III repeats.^17^ Since Caf1 also displays highly desirable properties (non‐adhesion, stability and ease of production) that are difficult to design de novo into protein polymers, we investigated whether it could be a useful animal‐free ECM substitute.

**Figure 1:**
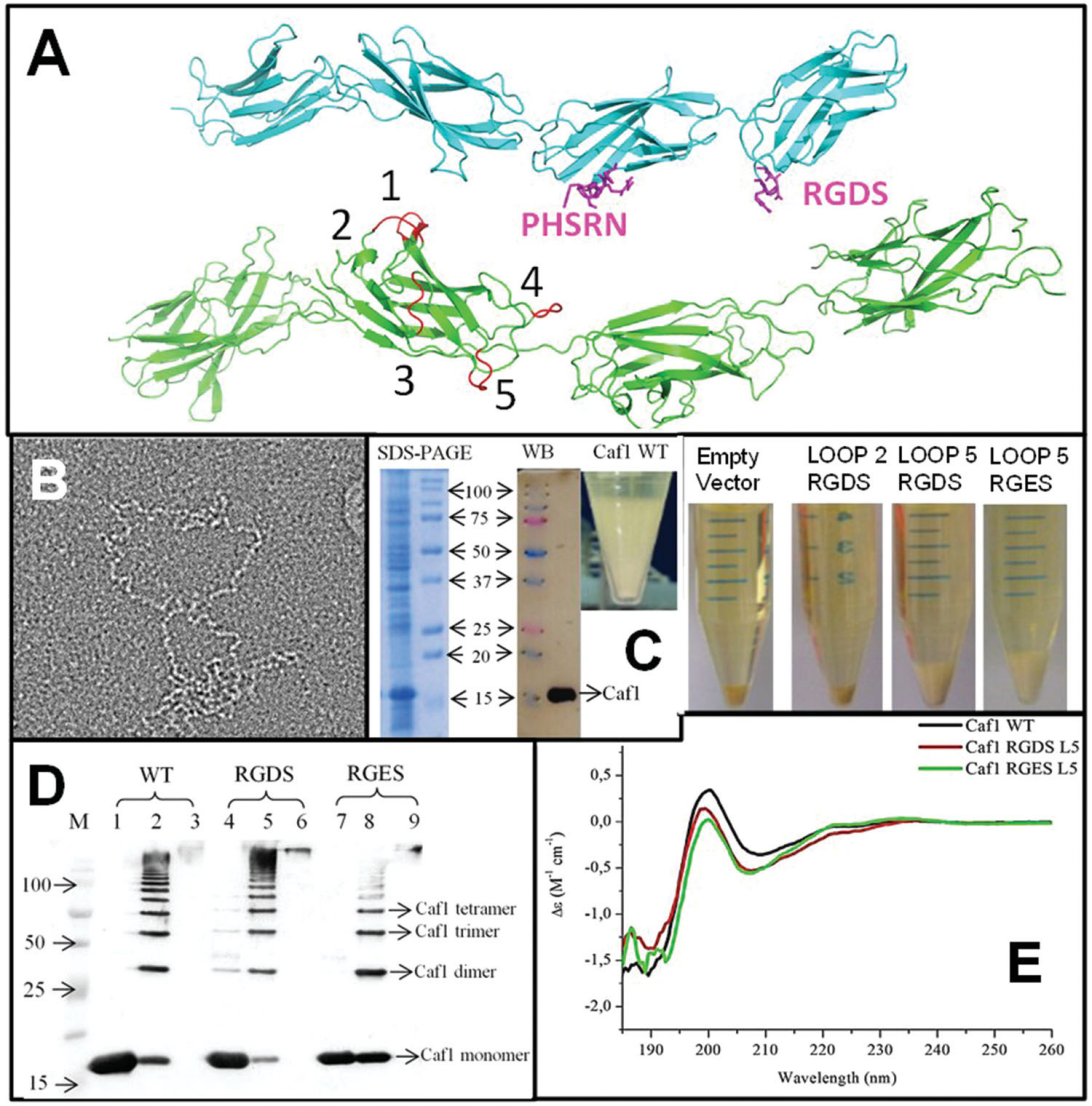
Expression of engineered Caf1. A) Upper molecule in cyan; Fibronectin Type III domain (PDB File 1FNF^17^ with sites of known cell adhesion motifs (RGDS and the accessory site PHSRN) highlighted in magenta. Lower molecule in green; Caf1 model based upon X‐ray and EM structures^10^ with RGDS insertion sites (loops) numbered. B) Linear, bead on a string, structure of Caf1 revealed by negative stain transmission electron microscopy. Box is 400 nm across. C) Expression of Caf1 showing pelleted cells and Caf1 rich flocculent layer. SDS‐PAGE Coomassie blue stained gel with heat treated flocculent layer sample (left) and M, molecular weight markers (molecular mass x 10^3^ kDa arrowed) (right). WB‐Western blot using anti‐Caf1 antibodies. Pelleted cells showing lack of flocculent layer in empty vector and Loop2 RGDS samples whilst a clear flocculent layer is present in the Loop 5 mutant tube. D) Analysis of purified Caf1 polymers by western blotting using a mouse monoclonal anti‐Caf1 antibody. WT (lanes 1–3), Loop5 RGDS (lanes 4–6) and Loop5 RGES (lanes 7–9). Lanes 1, 4, 7 heated at 95 °C for 5 min showing only monomeric Caf1. Lanes 2, 5, 8 heated at 95 °C for 45 s showing a ladder of Caf1 multimers. Lanes 3, 6, 9 unheated showing only high MW polymers. E) Far UV‐CD spectrum of WT, Loop5 RGDS and Loop5 RGES Caf1 polymers. Each curve represents the average of 10 accumulated spectra measured at a concentration of 0.5 mg mL^−1^ Caf1 (0.05 cm path length cell). The sample contained 50 mM sodium phosphate, pH 7.2. Each spectrum was corrected by subtraction of a comparable blank. The abscissa is in units Δε (M^−1^ cm^−1^) where M is the molar concentration of amino acid residues.

To imitate fibronectin, we inserted into Caf1 the Arg‐Gly‐Asp‐Ser peptide (RGDS) which has been shown to confer cell adhesion properties similar to the whole fibronectin molecule.^19^ The RGDS motif was incorporated into surface loops in order to expose the motif as in fibronectin,^17^ limit the changes to the structure of Caf1 and also to avoid important sites of Caf1: chaperone/usher interactions.^10^ Inserts were modeled using PyMOL^20^ and the published coordinates for Caf1 (PDB file: 1Z9S)^10^ and finally five mutants were expressed and purified (Figure 1A).

The expression of *caf1*, from its own temperature dependent promoter, was revealed by the presence of a flocculent layer (FL) above the cell pellet (CP) after centrifugation^14^ (Figure 1C). We analyzed this polymer rich layer by SDS‐PAGE using three methods of preparation, no heat denaturation in which the polymer is intact (Figure 1D lanes 3, 6, 9), limited heat denaturation (45 s at 95 °C) in which a ladder of oligomers is formed (Figure 1D lanes 2, 5, 8) and full heat denaturation (5 min at 95 °C) in which mostly monomers are observed^12^ (Figure 1D lanes 1,4,7). In the heat denatured sample a significant band ≈ 15 kDa was confirmed as Caf1 monomer by western blot and peptide mass finger printing (Supporting Information, Figure S1 and 2).

Of the five mutants, L2RGDS (Loop 2 insertion) did not express Caf1, L5RGDS had the highest yield and thus we constructed a loop 5 Caf1 RGES mutant (L5RGES) as a non‐cell‐adhesive control^21^ and a high yield of L5RGES was also obtained (Figure 1C). Both formed WT‐like polymers of high molecular weight (Figure 1D) and the thermal transition temperature of unfolding (Tm), obtained by Far‐UV CD (Figure 1E) and DSC, were little changed Caf1 WT (DSC = 86 °C, CD = 83 °C), Caf1 L5RGDS (84/83 °C), and Caf1 L5RGES (83/81 °C). The far‐UV CD spectra of Caf1 RGD/ES L5 show slight differences to that of Caf1WT. This is an unusual spectrum, quite unlike classic the beta‐strand structures, so the reasons for the weaker signal at 205 nm is unclear. Thus we checked the structure by near UVCD and electron microscopy. The spectra for near UV CD (Supporting Information, Figure S3A) for the Caf1 WT, Caf1 RGDS L5 and RGES L5 again show a similar structure. The main structural characteristics revealed on the near UV CD spectrum previously^12^ such as the two minor (at 262 and 269 nm) and major peaks at 283 and 290 nm were observed in this study. Analysis of transmission electron microscopy images of negatively stained Caf1 fibres was performed using Jmicrovision software.^22^ We determined the length of Caf1 WT, Caf1 RGDS L5, Caf1 RGES L5 fibres and the mean fiber lengths were 401, 323, and 257 nm, respectively (Supporting Information, Figure S3B). However it was evident, by inspecting the box charts, that there was a great heterogeneity of Caf1 fiber length in all samples (Supporting Information, Figure S3B). Similar results for Caf1 WT fiber length were observed by Soliakov and colleagues.^12^

Next, to assess Caf1's suitability for cell culture, cell viability was assayed via calcein uptake. When cultured on surfaces coated with Caf1‐RGDS, Caf1‐RGES and Caf1‐WT neither 3T3 fibroblasts nor PC12 cell lines revealed any toxicity within 48 h (Supporting Information, Figure S4). However, 3T3 cells proliferated more on fibronectin. Fibronectin is a large protein which contains several other important peptides for cell proliferation such as PHSRN, LDVP and IDAP and so the challenge is to incorporate several different motifs in Caf1 fibers as shown later.

We then studied cell morphology and adhesion in detail using scanning electron microscopy (SEM). Each cell line was grown on 12 mm glass cover slips pre‐coated with either Caf1WT, L5RGDS, L5RGES, Fibronectin or Collagen IV, incubated for 24 h, fixed and visualized by SEM (**Figures** **2**, **3**). Caf1WT inhibits adhesion of both PC12 and 3T3 cells whereas this effect is reversed on L5RGDS (Figure 2A and B). To quantify cell adhesion on Caf1 protein‐coated glass surfaces and on the control surfaces: collagen IV and fibronectin, ten images, of each cell line were examined. The total number of cells counted varied between 558 for PC12 on collagen IV to 9 for 3T3 on WT Caf1 and the percentage of cell adhesion was determined (Supporting Information, Table S2). This revealed that approximately 80% of 3T3 cells bound to the L5RGDS and fibronectin surfaces whilst only 3% bound to the WT Caf1 surface. However, the most striking differences were apparent when the shapes of the adherent cells were analyzed according to their interaction with the surface i.e., do they present one or more filopodia (cytoplasmic projections) (Figures 2 and 3). Thus Caf1WT provided a surface where the cells were invariably round and showed no projections. Critically, this behaviour of 3T3 and PC12 was largely reversed by the simple insertion of the RGDS motif, such that the results were identical to those on fibronectin (Figures 2 and 3 and Supporting Information, Figure S5). Whilst the results for both 3T3 and PC12 cells on L5RGDS mimic those of fibronectin, PC12 cells, as expected, grow rather better on collagen (Figure 3).

**Figure 2:**
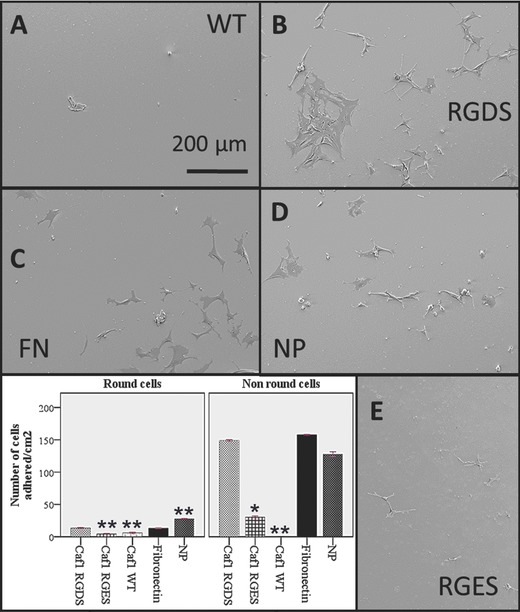
Fibroblasts on Caf1 polymers. Glass slides were incubated with each of the proteins shown then used to culture mouse 3T3 fibroblasts for 24 h before being finally fixed and imaged by scanning electron microscopy. A) WT Caf1 polymer. B) Loop 5 RGDS Caf1 polymer. C) Fibronectin. D) Buffer treated glass – no protein. E) Control Loop 5 RGES polymer. Histograms show differences in cell morphology. Non‐round cells show one or more filopodia. Data represent the mean of three experiments ± standard error of the mean (S.E.M). Significance was determined by one way ANOVA analysis with Scheffe as a post hoc test was conducted. (*) *P* < 0.01 compared to fibronectin, (**) *P* < 0.001 compared to fibronectin.

**Figure 3:**
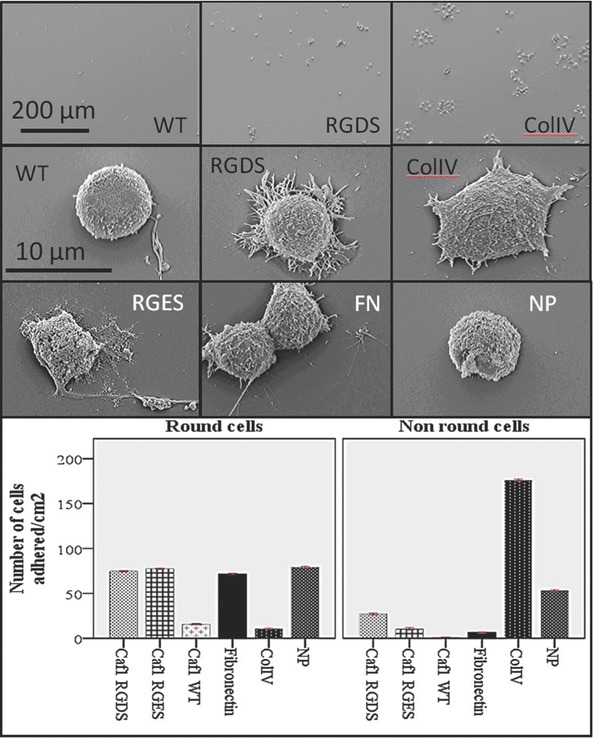
PC12 cells on Caf1 polymers. Glass slides were incubated with each of the proteins shown, used to culture rat pheochromocytoma PC12 cells for 24 h before being finally fixed and then imaged by scanning electron microscopy. Top; low magnification images to display differences in cell numbers on WT Caf1, Loop 5 RGDS Caf1 and collagen IV polymers. Lower images; comparison of cell morphology on the different polymers WT Caf1 polymer; Loop 5 RGDS Caf1 polymer; Collagen IV polymer; Loop 5 RGES polymer; Fibronectin (FN) and Buffer treated glass – no protein (NP) Histograms show differences in cell morphology. Non‐round cells show one or more filopodia. Data represent the mean of three experiments ± standard error of the mean (S.E.M). Significance was determined by one way ANOVA analysis with Scheffe as a post hoc test was conducted. All treatments were *P* < 0.001 compared to collagen IV.

Previous work on other CU proteins has shown that when two subunit genes are expressed in the same cell they combine in the same polymer.^23^ This offers a synthetic biology approach whereby a series of different monomers could be expressed in a single cell under the control of different promoters to create a range of mixed polymers on demand. To demonstrate this possibility for the Caf1 polymer system we expressed two different genes with two plasmids each carrying a different origin of replication, selection marker and promoter (**Figure** **4**A); pAH34L containing the complete wild type Caf1 operon^14^ and pBAD33^24^ which expressed only a mutant *caf1* gene which included a FLAG epitope termed *caf1‐FLAG*. The expression of *caf1* encoded by pAH34L is temperature sensitive whilst the expression levels of *caf1‐FLAG* encoded by pBAD33 can be modulated over a varied range of L‐arabinose concentrations. Analysis of co‐expression of Caf1 mutants was performed by western blot. Since the pBAD33 plasmid carries neither chaperone nor usher protein genes the caf1‐FLAG did not form polymers by itself (Figure 4C lane 6). As earlier shown in Figure 1D, the western blot in Figure 4B shows that WT pAH34L plasmid formed a polymeric protein. No full length polymer protein was detected by western blot in the unheated sample (Figure 4B lane 7) because transferring such large polymers to the nitrocellulose is inefficient. However, dimers and trimers can be seen in the sample heated for 45s (Figure 4B, lane 8) and only monomer is observed in the fully heat denatured sample (Figure 4B lane 9). Finally, co‐expression of the two plasmids produced FLAG labeled dimers and trimers when heated for 45s (Figure 4C lane 2). Thus, caf1‐FLAG must have assembled into polymers using the WT usher protein supplied by plasmid pAH34L. Since the corresponding lane 2 in Figure 4C also reacts with anti‐Caf1 antibodies it is thus reasonable to assume that we have produced mixed polymers composed of the products of both plasmids and not pure polymers of the different subunits. (Figure 4B and C lane 2). Thus, Caf1‐FLAG was detected outside the cell in the flocculent layer (Figure 4C) showing for the first time the export of Caf1 hybrid polymers. In future the scaffold could more closely mimic the complexity of ECM by containing several functional monomers including different cell adhesion motifs e.g., PHSRN (Figure 1A) or protease remodelling sites.

**Figure 4:**
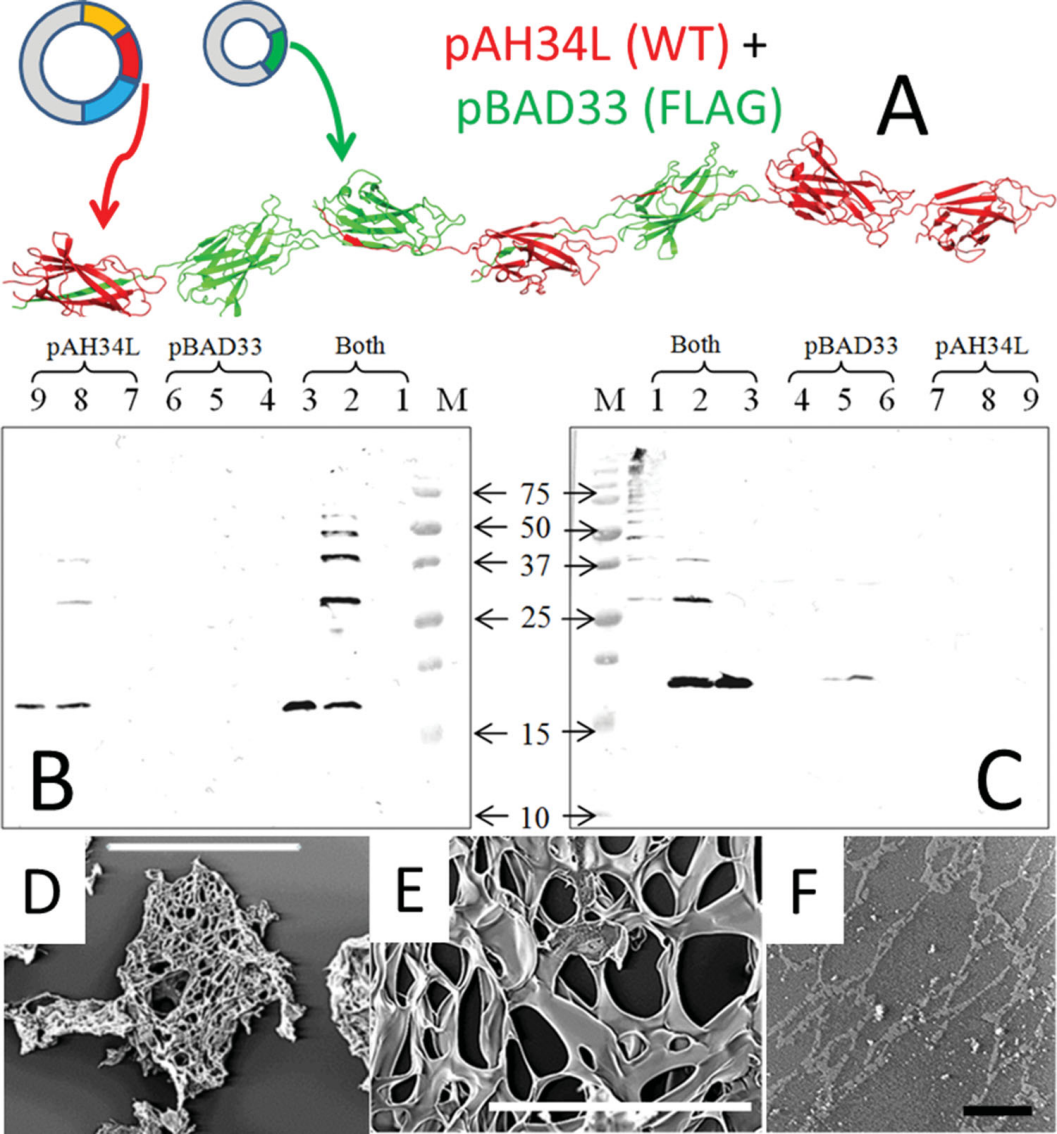
Complex polymers and hydrogels. A) Schematic of the co‐expression of WTCaf1 with Caf1‐FLAG using the plasmids pAH34L (WTCaf1, chaperone and usher genes) and pBAD33 (Caf1‐FLAG only). WT and mutant monomers expressed from different plasmids create mosaic Caf1 polymers composed of the two different subunit types. WTCaf1 represented in red and Caf1‐FLAG in green. (B) pBAD33_SD_caf1 NT‐FLAG + pAH34L probed with anti‐Caf1 antibody. M, molecular weight marker proteins (molecular mass kDa); lane 1, pBAD33_SD_caf1 NT‐FLAG + pAH34L sample non‐heated; lane 2, pBAD33_SD_caf1 NT‐FLAG + pAH34L sample heated at 95 °C for 45 seconds; lane 3, pBAD33_SD_caf1 NT‐FLAG + pAH34L sample heated at 95 °C for 5 minutes; lane 4, pBAD33_SD_caf1 NT‐FLAG sample non heated; lane 5, pBAD33_SD_caf1 NT‐FLAG sample heated at 95 °C for 45 seconds; lane 6, pBAD33_SD_caf1 NT‐FLAG sample heated at 95 ºC for 5 minutes; lane 7, pAH34L sample non‐heated; lane 8, pAH34L sample heated at 95 °C for 45 seconds; lane 9, pAH34L sample heated at 95 °C for 5 minutes. (C) Identical samples probed with anti‐Flag antibody. Lane 2 shows incorporation of Flag‐mutant from pBAD33 into Caf1 polymers. The monomer bands on Figure 4B and C have run at different levels, largely due to gel differences (see markers) but possibly also due to the introduction of the charged FLAG epitope D,E) SEM of freeze dried Caf1 hydrogel crosslinked with 4 arm PEG. Scale bar (D) = 500 μM and scale bar (E) = 50 μM. (F) ESEM of Caf1 hydrogel crosslinked with 4 arm PEG. Scale bar = 50 μM.

The Caf1 polymer can also be used to form a hydrogel with possible applications in 3D cell culture. WTCaf1 at a final concentration of 30 mg mL^−1^ produces a viscous solution which we stabilized by the addition of amine reactive cross linkers of various lengths DTSSP (12.0 Å), NHS‐PEG‐NHS (197 Å), and 4‐arm NHS‐PEG (2 × 197 Å). These can react with any of the eight surface lysines on each Caf1 monomer. The Caf1 hydrogels were characterized using a simple tube‐inversion assay in which non–cross linked solutions would flow down the sides of the plastic reaction vessel. The gelation time was visually estimated (Supporting Information, Table S3) to be within 24 to 27 min for NHS‐PEG‐NHS and 2 to 22 min for 4‐arm PEG‐NHS, depending on the concentration of the cross‐linker. The higher the concentration of these two cross‐linkers the quicker the gelation time. With DTSSP a solid gel was not observed. The increase in gelation rate of 4‐arm PEG‐NHS could be due to the structure of 4‐arm PEG which influences its ability to react with the primary amine groups of Caf1.^25^ The gelation time reported here is comparable with other studies using PEG hydrogels, for example Liu et al., (2012)^26^ who examined the combination of PEG diacrylate (PEGDA) and acryloyl‐PEG‐RGD.

After cross‐linking for 30 min, samples were analyzed on a 4–20% gradient gel. The band of approximately 15 kDa corresponding to the Caf1 monomer in the non‐cross linked Caf1 sample (control) was used as a reference for the subsequent analysis. The relative density of the unreacted Caf1 monomer band, in Caf1 samples cross‐linked with the different cross‐linkers at various concentrations, was determined. Since the calculations of the relative density for the high molecular weight bands were more complex, these were considered a single band and referred to as the “Caf1 cross‐linked fraction”. The 4‐arm PEG‐NHS showed the least amount of residual monomer and thus a higher degree of cross linking (Supporting Information, Table S4)

Caf1 polymers cross‐linked with a short arm length cross‐linker such as DTSSP (12.0 Å) promote a closer contact between the Caf1 fibres. Thus, the images obtained by TEM revealed a compact Caf1 hydrogel which reflects the Caf1 fibres proximity. A different result was obtained for Caf1 polymers cross‐linked with a long spacer NHS‐PEG‐NHS (197 Å), which can better separate the Caf1 fibres. Large Caf1 hydrogel meshes were observed by TEM. When we used the 4‐arm PEG‐NHS where each cross section is also 197 Å the interactions between Caf1 and the 4‐arm PEG‐NHS the structure was more condensed than that produced by NHS‐PEG‐NHS (Supporting Information, Figure S6).

When using a monomeric form of Caf1 made by circular permutation, cpCaf1,^13^ these large networks were not seen (Supporting Information, Figure S7). A highly porous hydrogel (Supporting Information, Figure S7) could be advantageous for swelling and water uptake and also as a scaffold for cell culture that can allow the passage of nutrients, oxygen through the pores. The TEM images confirmed that the formation of the Caf1 hydrogels depends on the cross‐linker concentration and structure.

The pore diameters of 4‐arm PEG‐NHS stabilized Caf1 hydrogels were assessed by SEM and ESEM (environmental SEM).^27^ For SEM we used freeze‐dried hydrogels which broke into fragments during analysis (Figure 4D) and revealed a mean pore diameter of 8 ± 1.9 μm (Figure 4E). ESEM analysis avoids the dehydration process and these images revealed a mesh‐like network structure with a mean pore diameter of 300 ± 0.3 nm (Figure 4F).

As shown by Lutolf and Hubbell (2005)^3^ in tight gel networks, cells are required to use proteolytic strategies to degrade the surrounding matrix and be able to migrate through the gel. Cells can remain viable in stiffer gels, however they cannot spread and proliferate which is fundamental for obtaining cell‐cell contacts and subsequent tissue formation.^28^ The Caf1 polymer thus can be made to imitate different ECM by having varied sub‐unit composition, different crosslinking ratios and possible protease cleavage sites. Its ease of production in an inexpensive and safe host bacterium means that it is a viable bulk product able to replace expensive cell culture reagents.

## Experimental Section

For details of the experimental methods used please see the Supporting Information.

## Supplementary Material

As a service to our authors and readers, this journal provides supporting information supplied by the authors. Such materials are peer reviewed and may be re‐organized for online delivery, but are not copy‐edited or typeset. Technical support issues arising from supporting information (other than missing files) should be addressed to the authors.

Supplementary

## References

[1] F. Rosso, G. Marino, A. Giordano, M. Barbarisi, D. Parmeggiani, A. Barbarisi, Journal of Cellular Physiology 2005, 203, 465 15744740 10.1002/jcp.20270

[2] a) K. Bott, Z. Upton, K. Schrobback, M. Ehrbar, J. A. Hubbell, M. P. Lutolf, S. C. Rizzi, Biomaterials 2010, 31, 8454; 20684983 10.1016/j.biomaterials.2010.07.046

[3] M. P. Lutolf, J. A. Hubbell, Nature Biotechnology 2005, 23, 47 10.1038/nbt1055 15637621

[4] Y. D. Du, R. Rosqvist, A. Forsberg, Infection and Immunity 2002, 70, 1453 11854232 10.1128/IAI.70.3.1453-1460.2002 PMC127752

[5] F. G. Sauer, H. Remaut, S. J. Hultgren, G. Waksman, Biochim. Biophys. Acta ‐ Mol. Cell Res. 2004, 1694, 259 10.1016/j.bbamcr.2004.02.010 15546670

[6] E. E. Galyov, A. V. Karlishev, T. V. Chernovskaya, D. A. Dolgikh, O. Y. Smirnov, K. I. Volkovoy, V. M. Abramov, V. P. Zavyalov, FEBS Letters 1991, 286, 79 1677900 10.1016/0014-5793(91)80945-y

[7] S. Geibel, E. Procko, S. J. Hultgren, D. Baker, G. Waksman, Nature 2013, 496, 243 23579681 10.1038/nature12007 PMC3673227

[8] A. Busch, G. Waksman, Philosophical Transactions of the Royal Society B ‐ Biological Sciences 2012, 367, 1112 10.1098/rstb.2011.0206 PMC3297437 22411982

[9] F. Sebbane, C. Jarrett, D. Gardner, D. Long, B. J. Hinnebusch, Infection and Immunity 2009, 77, 1222 19103769 10.1128/IAI.00950-08 PMC2643634

[10] A. V. Zavialov, J. Berglund, A. F. Pudney, L. J. Fooks, T. M. Ibrahim, S. MacIntyre, S. D. Knight, Cell 2003, 113, 587 12787500 10.1016/s0092-8674(03)00351-9

[11] A. V. Zavialov, V. M. Tischenko, L. J. Fooks, B. O. Brandsdal, J. Aqvist, V. P. Zav'Yalov, S. MacIntyre, S. D. Knight, Biochem. J. 2005, 389, 685 15799718 10.1042/BJ20050426 PMC1180718

[12] A. Soliakov, J. R. Harris, A. Watkinson, J. H. Lakey, Vaccine 2010, 28, 5746 20600492 10.1016/j.vaccine.2010.05.074

[13] D. A. Chalton, J. A. Musson, H. Flick‐Smith, N. Walker, A. McGregor, H. K. Lamb, E. D. Williamson, J. Miller, J. H. Robinson, J. H. Lakey, Infection and Immunity 2006, 74, 6624 16982834 10.1128/IAI.00437-06 PMC1698084

[14] J. Miller, E. D. Willamson, J. H. Lakey, M. J. Pearce, S. M. Jones, R. W. Titball, FEMS Immunol. Med. Microbiol. 1998, 21, 213 9718211 10.1111/j.1574-695X.1998.tb01168.x

[15] L. Vitagliano, A. Ruggiero, C. Pedone, R. Berisio, Biochemical and Biophysical Research Communications 2008, 372, 804 18534189 10.1016/j.bbrc.2008.05.145

[16] O. Salih, H. Remaut, G. Waksman, E. V. Orlova, Journal of Molecular Biology 2008, 379, 174 18448124 10.1016/j.jmb.2008.03.056

[17] D. J. Leahy, I. Aukhil, H. P. Erickson, Cell 1996, 84, 155 8548820 10.1016/s0092-8674(00)81002-8

[18] E. Oezkan, R. A. Carrillo, C. L. Eastman, R. Weiszmann, D. Waghray, K. G. Johnson, K. Zinn, S. E. Celniker, K. C. Garcia, Cell 2013, 154, 228 23827685 10.1016/j.cell.2013.06.006 PMC3756661

[19] M. D. Pierschbacher, E. Ruoslahti, Nature 1984, 309, 30 6325925 10.1038/309030a0

[20] The PyMOL Molecular Graphics System *Schrödinger, LLC* 2010

[21] U. Hersel, C. Dahmen, H. Kessler, Biomaterials 2003, 24, 4385 12922151 10.1016/s0142-9612(03)00343-0

[22] N. Roduit, Vol. Version 1.2.2, 2007

[23] M. Baga, M. Norgren, S. Normark, Cell 1987, 49, 241 2882856 10.1016/0092-8674(87)90565-4

[24] L. M. Guzman, D. Belin, M. J. Carson, J. Beckwith, Journal of Bacteriology 1995, 177, 4121 7608087 10.1128/jb.177.14.4121-4130.1995 PMC177145

[25] H. Tan, A. J. DeFail, J. P. Rubin, C. R. Chu, K. G. Marra, Journal of Biomedical Materials Research Part A 2010, 92A, 979 19291691 10.1002/jbm.a.32438 PMC2811768

[26] Z. Liu, L. Xiao, B. Xu, Y. Zhang, A. F. T. Mak, Y. Li, W.‐y. Man, M. Yang, Biomicrofluidics 2012, 6 10.1063/1.4704522 PMC3338548 22550556

[27] F. M. Plieva, M. Karlsson, M. R. Aguilar, D. Gomez, S. Mikhalovsky, I. Y. Galaev, Soft Matter 2005, 1, 303 10.1039/b510010k 32646121

[28] C. S. Chen, J. Tan, J. Tien, Annual Review of Biomedical Engineering 2004, 6, 275 10.1146/annurev.bioeng.6.040803.140040 15255771

